# Chitosan nanoparticle-based neuronal membrane sealing and neuroprotection following acrolein-induced cell injury

**DOI:** 10.1186/1754-1611-4-2

**Published:** 2010-01-29

**Authors:** Youngnam Cho, Riyi Shi, Richard Ben Borgens

**Affiliations:** 1 Center for Paralysis Research, School of Veterinary Medicine, Purdue University, West Lafayette, IN 47907, USA; 2 Weldon School of Biomedical Engineering, Purdue University, West Lafayette, IN 47907, USA

## Abstract

**Background:**

The highly reactive aldehyde acrolein is a very potent endogenous toxin with a long half-life. Acrolein is produced within cells after insult, and is a central player in slow and progressive "secondary injury" cascades. Indeed, acrolein-biomolecule complexes formed by cross-linking with proteins and DNA are associated with a number of pathologies, especially central nervous system (CNS) trauma and neurodegenerative diseases. Hydralazine is capable of inhibiting or reducing acrolein-induced damage. However, since hydralazine's principle activity is to reduce blood pressure as a common anti-hypertension drug, the possible problems encountered when applied to hypotensive trauma victims have led us to explore alternative approaches. This study aims to evaluate such an alternative - a chitosan nanoparticle-based therapeutic system.

**Results:**

Hydralazine-loaded chitosan nanoparticles were prepared using different types of polyanions and characterized for particle size, morphology, zeta potential value, and the efficiency of hydralazine entrapment and release. Hydralazine-loaded chitosan nanoparticles ranged in size from 300 nm to 350 nm in diameter, and with a tunable, or adjustable, surface charge.

**Conclusions:**

We evaluated the utility of chitosan nanoparticles with an in-vitro model of acrolein-mediated cell injury using PC -12 cells. The particles effectively, and statistically, reduced damage to membrane integrity, secondary oxidative stress, and lipid peroxidation. This study suggests that a chitosan nanoparticle-based therapy to interfere with "secondary" injury may be possible.

## Background

Substantial cellular injury caused by acute mechanical, chemical, or biological insult is initially associated with an instantaneous loss of plasma membrane integrity. This facilitates the unregulated exchange of intracellular/extracellular ion species, and subsequently leads to cell death [1,2]. The failure of this functional barrier further induces incomplete oxygen metabolism within the cell, and accelerates the production of highly reactive oxygen species (so-called "free radicals") such as superoxide anions, hydrogen peroxide, and hydroxyl radicals. The biochemical reactivity of such reactive oxygen species (ROS) triggers the deterioration of lipids within the inner domain of the cell membrane (lipid peroxidation or LPO) producing endogenous toxins composed of mainly aldehydes - especially acrolein [3-7]. Unfortunately, the continuing production of toxic aldehydes feed back to attack mitochondria, yet untouched healthy membrane, and pass through intact cell membranes to attack even nearby healthy cells. As a consequence, the initial loss of membrane integrity in response to the "primary injury" is the crucial step initiating a "secondary injury" process in the nervous system. Unchecked, these self - reinforcing processes influence further collapse of mitochondria and oxidative metabolism, continued deterioration of the cell membrane, the further production of endogenous toxins, and ultimately, cell death. In the past decade, significant neuroprotection was achieved by the topical, intravenous, or even subcutaneous application of water-soluble polymers, such as poloxamer 188, 1100, or polyethylene glycol (PEG), as a rescue strategy to alleviate cell and tissue damage following traumatic insults [8-16]. Such versatile polymers have demonstrated their capability to induce functional recovery at the cell level by initiating spontaneous molecular rearrangement of the lipid bilayers through membrane fusion and integration [13].

This membrane-based recovery is initially seen as an erasure of defects in the axolemma whose integrity is the basis for action potential conduction. Measurement of a rapid (minutes) recovery of compound action potentials traversing crushed or cut guinea pig spinal cord in organ culture [14,17] and an equally rapid recovery of somatosensory evoked potentials traversing the spinal cord lesion in "whole animal" guinea pig injury models reveals the initial consequences of acute neuroprotection/neurorepair by direct application - or intravenous injection - of these polymers [6]. The longer term, and stable, repair of critical anatomy has been documented by intracellular tracing in severely injured guinea pig spinal cord and adult rat brain [18,19]. This in turn is responsible for significant behavioral recovery of spinal cord and brain - mediated animal behavior [12,20,21]. PEG and its derivatives have preferable molecular weights and concentration ranges enabling them to carry out this neuroprotection, which significantly narrows the therapeutic window, and possibly their efficacy without side effects in clinical trials [22]. For example, higher concentration of PEG dissolved in sterile saline produce viscosity problems for injectable solutions whereas low molecular weight PEGs (< ~1000 kD) may produce ethylene glycol poisoning [23,24]. Additionally, it has been observed that bulky chains of poly (ethylene oxide) tend to inhibit the efficient entry of such synthetic polymers into cells where continued beneficial activity is desired. According to a previous study, PEG was accumulated only on the outside of cells for hours instead of internalizing, where it can produce other beneficial effects by acting on damaged mitochondria [25].

**Figure 1:**
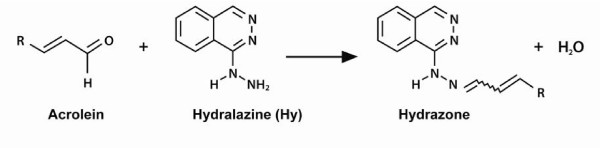
**The schematic illustration of nucleophile drug hydralazine and acrolein to form hydrazone which mediates acrolein toxicity formed during lipid peroxidation**. As a highly reactive α, β-unsaturated aldehyde, acrolein readily attacks nucleophilic centers of cell proteins and DNA by Michael addition chemistry. Such intracellular toxicity induced by acrolein causes progressive destructive secondary reactions, disrupting molecular and cellular systems. Scavenging of hydralazine inhibits acrolein-mediated toxicity and deactivates reactive carbonyl adducts via the formation of hydrazone.

Nanoparticle-based drug delivery in which an acrolein-trapping agent (hydralazine) is loaded within the construction would be one of the ways to resolve these problems. We have observed anatomical and functional recovery of axonal damage in guinea pig spinal cord after intravenous injections of a simple solution of chitosan in sterile saline. Indeed chitosan possess "sealing" qualities similar to PEG and the Poloxamers (unpublished data to appear elsewhere). Since it is now well-established that the inactivation of acrolein can be achieved by the formation of hydrazone adjuncts on the basis of interaction between acrolein and its trapping agent, hydralazine (Figure 1), we investigated the potential *dual *benefit by constructing a chitosan nanoparticle acting as 1., a membrane fusogen, and 2., a hydralazine delivery vehicle to ameliorate the damaging effects of acrolein exposure.

## Results

### Synthesis and characterization of hydralazine-loaded chitosan nanoparticles

**Figure 2:**
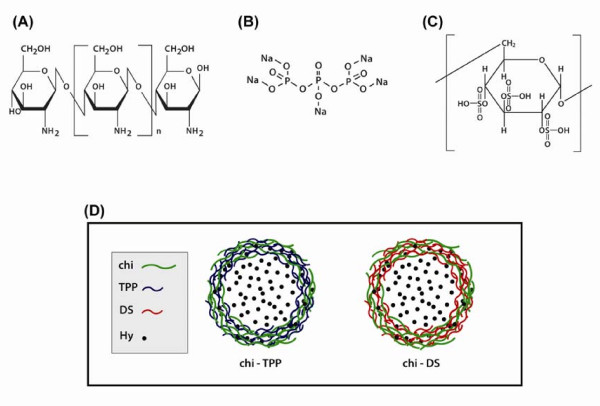
**Chemical structures of (A) chitosan (Chi), (B) sodium tripolyphosphate (TPP), and (C) dextran sulfate (DS)**. (D) The schematic illustration shows hydralazine incorporation into Chi-TPP and Chi-DS nanoparticles. The chitosan nanoparticles were formed spontaneously by mixing an equivalent concentration of Chi and polyanions, TPP or DS. To achieve the incorporation of hydralazine into nanoparticles, hydralazine was added in the prepared solution at a concentration of 1 mg/mL.

The molecular structures of chitosan, TPP, and DS are shown in (Figure 2). The preparation of chitosan nanoparticles was conducted by adopting well-established protocols [26-28]. The electrostatic interaction of positively charged amine moieties in hydralazine and chemically available functional groups of polyanions, such as phosphoric acid in TPP and the sulfate group in DS, is critical to facilitate the encapsulation of hydralazine inside chitosan nanoparticles. The morphological or physical phenomena of hydralazine encapsulation within nanoparticles were characterized by TEM and zeta potential/particle size analyzer as described above. In contrast to solid particles, the chitosan particles are not clearly identified once inside the cell due to some particle-formed aggregates which appeared as large agglomerates. We plan to work next with flow cytometry that could perhaps offer a better opportunity to observe the internalization of fluorescent-conjugated chitosan nanoparticles.

**Figure 3:**
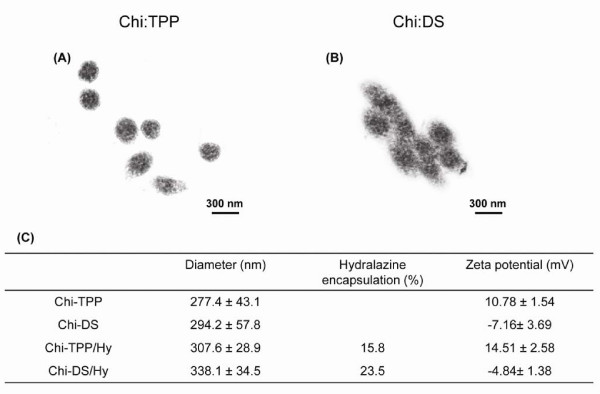
**The characterization of chitosan particles**. TEM images of (A) chitosan nanoparticles incorporating TPP and hydralazine (Chi-TPP/Hy) and (B) chitosan nanoparticles incorporating DS and hydralazine (Chi-DS/Hy). (C) provides data on the particle size, encapsulation efficacy, and zeta potential for chitosan nanoparticle formulations. The encapsulation efficiencies showed a range from 15% to 23%, depending on the types of polyanions. Mean diameter and surface charge of particles were slightly increased upon the incorporation of hydralazine due to the presence of positively charged moieties.

In all experiments, chitosan nanoparticles were formed at an equivalent mass ratio of chitosan to polyanion due to the fact that high or low concentrations of chitosan compared to polyanions tends to decrease the encapsulation efficiency and/or promote aggregation of particles [29]. Unloaded chitosan nanoparticles were measured to have diameters in the range of ~250 nm ~300 nm (Figure 3C). The incorporation of hydralazine caused a slight increase in the mean diameter of chitosan nanoparticles, resulting in an approximately 300 nm ~350 nm range in diameter. The surface charge of unloaded chitosan nanoparticles ranged from 10.78 ± 1.54 mV to -7.16 ± 3.69 mV for Chi-TPP and Chi-DS, respectively. The number of negatively charged groups of the polyanions, TPP and DS, was responsible for this difference, where DS (MW 9,000 ~ 20,000 Da) would possess the predominant amount of sulfate groups per mole compared to the amounts of phosphoric acid of TPP (MW 368 Da) at experimental conditions (pH 3~4). Positively charged hydralazine loading did slightly increase these values, corresponding to 14.51 ± 2.58 mV and -4.84 ± 1.38 mV for Chi-TPP/Hy and Chi-DS/Hy, respectively. Analysis of particle morphology revealed that Chi-TPP nanoparticles exhibited a well-defined spherical shape with a solid and consistent structure (Figure 3A). On the contrary, Chi-DS nanoparticles showed a clustered spherical structure (Figure 3B). The opposite charge of hydralazine and polyanions significantly contributes to concomitant increase in the efficiency of encapsulation, resulting in 15.8% and 23.5% for Chi-TPP/Hy and Chi-DS/Hy, respectively. It is worthy to note that hydralazine entrapment was increased by approximately 35% due to the association with DS compared to TPP. This is possibly attributed to the presence of sufficient negative charge densities in DS, which facilitated the encapsulation of appreciable quantities of hydralazine through the complexation process with chitosan.

### In vitro release of hydralazine

**Figure 4:**
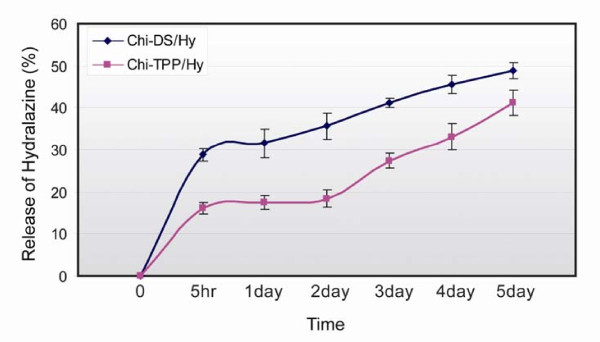
**The in vitro release profile of hydralazine from chi-TPP and chi-DS in Krebs' solution**. In both types of particles, initial burst release occurred, yet subsequently fell to a constant and slow release observed over 5 days.

The release profile of hydralazine from Chi-TPP and Chi-DS nanoparticles was observed in Krebs' solution over 5 days duration. Apparently, both types of particles showed burst release of 15 ~ 30% of the total determined concentration at 5 hr, which declined to a constant, but slow, release rate over several days, as shown in (Figure 4). It was likely that the rapid release was caused by desorption of hydralazine loosely attached to the surface of chitosan nanoparticles. The relative small size of the hydralazine molecule would not be interfered by the diffusion/dissociation process stemming from the core or the pores of nanoparticles. In addition, the high affinity and hydrophilic nature of chitosan with Kreb's medium provides space for penetration inside the particles to be able to dissolve the entrapped drug. It should be noted that the molecular size of drug and ionic interaction with polyanions would be a major consideration in deciding the rate of drug release.

### The effect of hydralazine-loaded chitosan nanoparticles on acrolein-mediated cell injury

**Figure 5:**
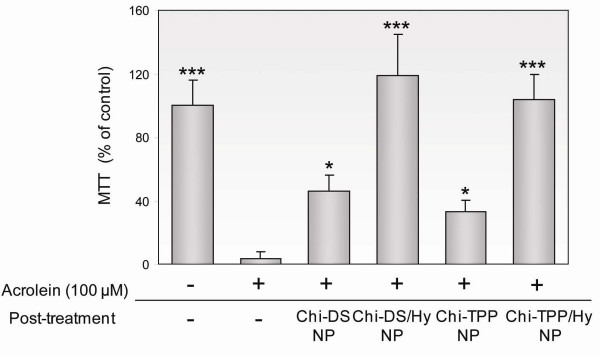
**Cell viability test by MTT assay to demonstrate the recovery of mitochondria functionality**. After 15 min of acrolein-mediated attack, cells were exposed to Chi-TPP and Chi-DS incorporated with or without hydralazine. Direct localization of hydralazine released from chitosan particles was observed, resulting in significantly enhanced viability. Results are expressed as percent control values ± SD (*n *= 5). *P < 0.05, ***P < 0.001.

Initially, functional tests (MTT and LDH exclusion tests) were used to evaluate the response to different chitosan formulations in PC 12 cells suffering from acrolein exposure. We compared the effectiveness of these formulations using entire cell populations in six different groups: 1) a control group, 2) a 100 μM acrolein-exposed group, 3) a 100 μM acrolein-exposed and Chi-DS nanoparticle - treated group, 4) a 100 μM acrolein-exposed and Chi-DS/Hy nanoparticle - treated group, 5) a 100 μM acrolein-exposed and Chi-TPP nanoparticle - treated group, and 6) a 100 μM acrolein-exposed and Chi-TPP/Hy nanoparticle - treated group. First, we tested whether chitosan nanoparticles were capable of sufficiently reducing acrolein cytotoxicity using the MTT assay (Figure 5). In all experiments, the various types of nanoparticles were added to the cell medium after 15 min following the exposure to acrolein. Acrolein-induced cytotoxicity was expressed as a percentage of MTT activity, which is, as explained above, an indicator of aberrant mitochondria functioning. Thus, MTT activity subsequent to 100 μM acrolein exposure was decreased to 3.5 ± 2.9% of controls values. In contrast, the treatment of poisoned cells with Chi-TPP or Chi-DS increased survival to 44.5 ± 12.6%, and 37.8 ± 9.6% of controls after 5 hr, respectively (P < 0.05). Even more significant increase was observed after application of hydralazine-entrapped chitosan nanoparticles. Treatment with Chi-DS/Hy or Chi-TPP/Hy showed very significant increase in cell viability, corresponding to 120.5 ± 26.6% and 108.6 ± 17.1% of control values, respectively (P < 0.001). Hydralazine-loaded chitosan nanoparticles improved cellular oxidative metabolism to the greatest extent - and even better than control value.

**Figure 6:**
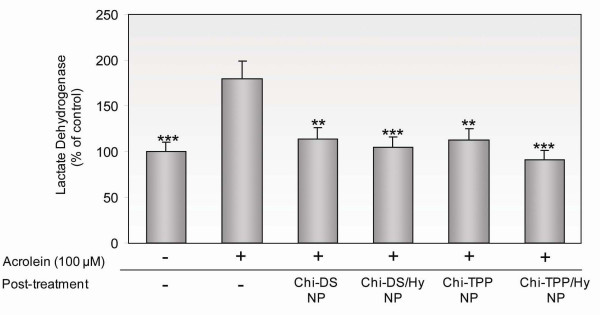
**Membrane permeability test by LDH assay to demonstrate the effects of acrolein and chitosan nanoparticles post-treatment**. LDH release was measured 3 hr after exposure to 100 μM acrolein followed by post-treatment with various chitosan nanoparticles. Chitosan nanoparticles were demonstrated to be capable of restoring plasma membrane integrity, regardless of the presence of hydralazine. Results are expressed as percent control values ± SD (*n *= 5). One-way paired ANOVA and Post Hoc Newman Keul's test were used for statistical analysis. **P < 0.01, ***P < 0.001.

**Figure 7:**
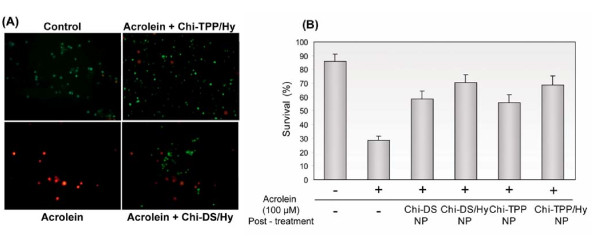
**Live/dead cell staining and the quantification of cell survival**. (A) Representative fluorescent images of PC 12 cells, approximately 15 minutes after acrolein challenge (100 μM). Cells were exposed to Chi-TPP/Hy and Chi-DS/Hy, respectively. (B) PC 12 cells survival rate was assessed as a ratio of live cells to total cells. Consistent with previous assays, survival was significantly increased as a result of the application of both types of chitosan nanoparticles. Results are expressed as percent control values ± SD (*n *= 5).

As a complementary test, we observed the release of LDH from PC 12 cells following acrolein attack. LDH results were essentially consistent with the values obtained from MTT assays. The cells exposed to 100 μM acrolein caused significant LDH increase, reaching 178 ± 25.4% of control values (Figure 6). Conversely, LDH release was reduced to 117 ± 18% and 115 ± 10% of control values by post-treatment with Chi-DS or Chi-TPP, respectively (P < 0.01). Remarkably, hydralazine-loaded chitosan nanoparticles, Chi-DS/Hy and Chi-TPP/Hy, caused even more reduction in LDH release to a level close to - or even below that of controls (untreated cells; 100 ± 2% of control values), corresponding to 105 ± 18% and 96 ± 19% of control values, respectively (P < 0.001). Additionally, we examined cell mortality using the live-dead cell assay. Consistent with all results described above, control cells in culture (with a characteristic 90 ± 7% survival) dramatically fell to 29.8 ± 4% at 5 hr when exposed to acrolein. Treatment of these poisoned cultures with different types of chitosan nanoparticles improved survival to 60 ~ 70% (Figure 7).

### The inhibition of plasma membrane peroxidation by the application of hydralazine-loaded chitosan nanoparticle

**Figure 8:**
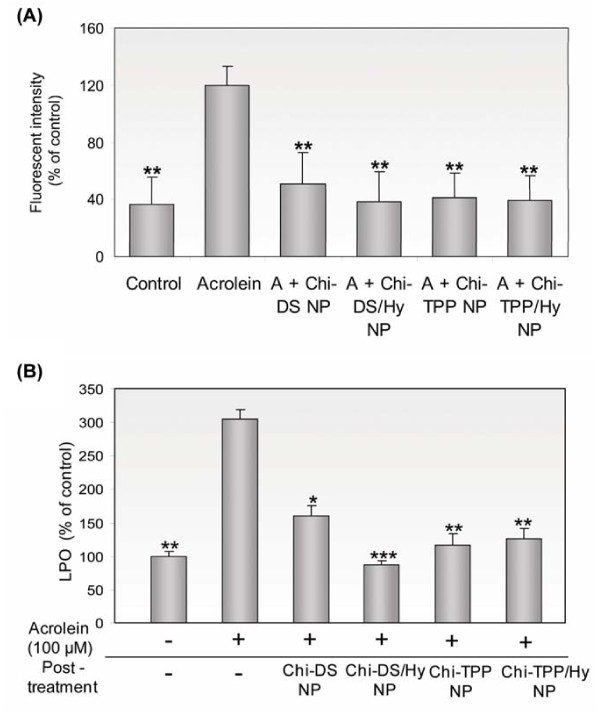
**ROS and LPO upon the exposure to acrolein, and inhibition by various types of chitosan nanoparticles**. (A) Quantification of oxidized HE fluorescence intensity of control, acrolein-induced injury, and acrolein followed by chitosan nanoparticle treatment of PC 12 cell groups (n = 5, **P < 0.01). (B) Measurements in control, acrolein-induced injured, and acrolein followed by chitosan nanoparticles treated PC 12 cell groups (n = 5, * P < 0.05, **P < 0.01, ***P < 0.001). The addition of chitosan nanoparticles following the exposure of acrolein showed markedly decreased rate of oxidation of membrane lipids as well as lipid peroxidation.

The addition of hydralazine-loaded chitosan nanoparticles to acrolein poisoned cultures was able to inhibit or interfere with the generation of ROS and the associated process of membrane LPO. The levels of ROS increased approximately four-fold after exposure of cells to 100 μM acrolein, corresponding to 120 ± 14% of control values (37 ± 19% (Figure 8A). Remarkably, the addition of chitosan nanoparticles reduced the level of ROS up to 39 ± 14% of control values - indeed close to that of control groups (38 ± 21%, P < 0.01). Consistent with this striking result, the level of LPO was also significantly increased to 305 ± 22% of control values after acrolein-induced injury (Figure 8B). Also consistent with previous results, the LPO level corresponded to 91 ± 5% (P < 0.001) of control values upon treatment with Chi-DS/Hy, which was even lower than that of the control group.

## Discussion

### Chitosan as a membrane "fusogen"

**Figure 9:**
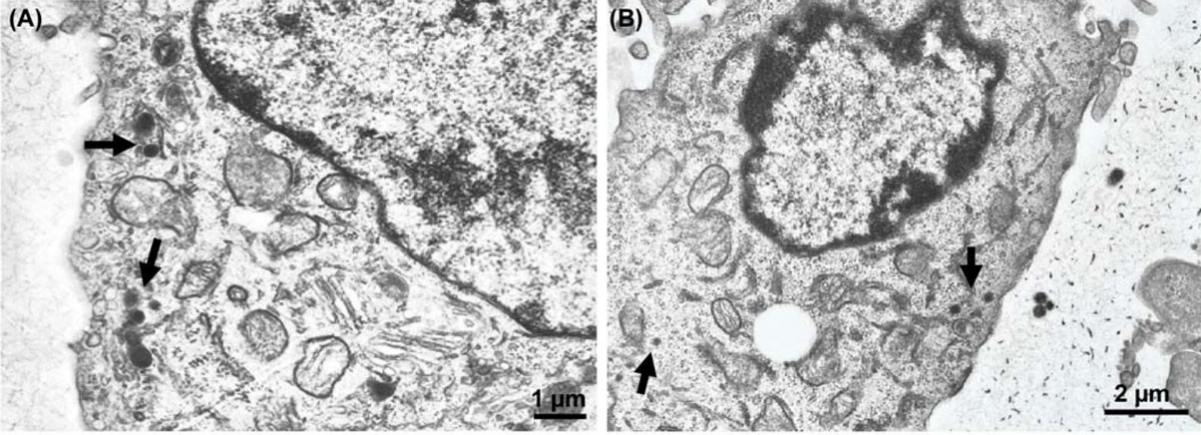
**Electron photomicrographs of (A) chi-DS and (B) chi-TPP cellular uptake by PC 12 neuronal cells**. The arrows indicate the presence of chitosan particles in PC 12 cells after incubation for 2 hours.

Chitosan, a biocompatible and biodegradable polymer, has been widely tested in a variety of fields for the purpose of developing treatments as diverse as wound healing, lung surfactant additives, and tissue engineering [30-32]. Additionally, substantial efforts have been devoted for the development and application of chitosan nanoparticles as vehicles for drug delivery [26,33-35]. In this regard, therapeutic efficacy based on chitosan nanoparticle systems confers several advantages: i) nanospheres composed of highly concentrated chitosan molecules could serve as a "fusogen". Previous studies have demonstrated the effect of chitosan derivatives on the fusion of small dipalmitoyl phosphatidylcholine (DPPC) bilayers by inducing the formation of large phospholipid aggregates [36-38], ii) in contrast to better known fusogens including PEG and Poloxamers, chitosan nanoparticles of small size and hydrophilic character are also able to cross the cell membrane to deliver drugs or other molecules to the cytosol while avoiding the rapid uptake by the reticuloendothelial system (RES), as shown in (Figure 9) [39]. It is particularly important that drugs carried inside nanoparticles would be protected from denaturing, enzymatic degradation, or the general biochemical environment, and iii) the encapsulation and release of drugs would be controllable by manipulating several characteristics of the particles such as size, surface functionalization, and loading concentration. Moreover, the preference of the chitosan moiety to target compromised membranes as discussed above further shows the feasibility for the selective localization of a large amount of the drug at a specific and desired site.

Recently, we have learned that chitosan, as a component of an injectable solution, can serve as a novel therapeutic agent after severe compression injury of guinea pig spinal cord (unpublished observations). In such injuries, progressive destruction of cells and tissues occurs after mechanical trauma, however critical anatomy and function was restored by the injection of chitosan. Chitosan accumulation typically occurs around the defect area in cells and tissues by hydrophobic interactions. Conversely, at intact membranes, high surface densities of lipid moieties inhibit the penetration of chitosan [36-38]. The mechanism underlying chitosan-mediated membrane sealing and specific targeting of injured membranes is still being investigated; however a clear demonstration of the latter observation is significantly persuasive that this approach holds unexpected merit in dealing with cell and tissue trauma.

The evidence presented in this report suggests the potential usefulness of chitosan - as a nanoparticle base likely due to its two-pronged attack on cell damage. We emphasize that chitosan nanoparticles themselves are capable of restoring cell viability by first mediating the sealing of damaged membrane. We additionally showed the application of chitosan achieved neuroprotection by interfering with the generation of ROS and LPO. This is also due to its membrane reconstruction properties rather than an ability to directly scavenge these toxins. However upon conjugation with hydralazine, its potential therapeutic effects are dramatically enhanced. Chitosan treatment alone did not provide neuroprotection after exposure to acrolein - even in high concentrations (unpublished observations). This fact suggested the inclusion of hydralazine in the chitosan nanoparticle to directly provide this function.

### Acrolein-induced progressive neuronal degeneration and rescue by hydralazine

It is widely accepted that traumatic injuries often deteriorate cell membrane integrity through multiple mechanisms involving several biological processes and biochemistries. Steady and progressive membrane rupture and concomitant loss of intracellular contents initially causes serious derangement of ionic species. Especially, the unregulated influx of Ca^2+ ^into cytoplasm plays a key role in destabilizing and collapsing the cytoarchitecture as well as membrane integrity by activating numerous catabolic physiological and biochemical processes such as ROS mediated LPO. There is little evidence that free radicals (with half lives less than seconds - even fractions of a second) are directly toxic to cells. It is the downstream end product of ROS stimulated LPO that produces actual toxins such as a variety of aldehydes - acrolein being the most potent. Compared to free radicals, acrolein has a half-life many orders of magnitude longer [40]. Indeed, highly reactive acrolein actively forms biomolecule conjugates with proteins, DNA, and glutathione, which further stimulates the generation of ROS and LPO. The cytotoxicity of acrolein is known to be concentration-dependent. After exposure to 75 - 100 μM acroleoin, a majority of the PC 12 cell population was dead within 4 hr [41]. In previous reports, we have shown that the toxicity of acrolein can be significantly diminished after application of an acrolein-trapping agent such as hydralazine. Hydralazine, a well-known nucleophile drug, is able to immediately inhibit acrolein cytotoxicity by forming "hydrazones" through a scavenging reaction (Figure 1) [42-44].

### Notes on the fabrication, characterization, and use of hydralazine-loaded chitosan nanoparticles in CNS trauma and neurodegenerative diseases

There are many studies to take advantage of colloidal particles as drug carriers. The use of colloidal drug carriers can facilitate not only site-specific drug delivery in a designated region but also facilitate drug bioavailability and therapeutic efficacy. On the basis of these considerations, a variety of biocompatible and biodegradable materials such as synthetic or natural polymers, lipids, or solid (metal, semiconductor, magnetic, or insulator) components have been intensively investigated [45,46].

First, liposome-based colloidal carriers have already commercialized. Despite these advances, liposomes still have some technical weakness with regard to the limitations in reproducibility, stability, and low drug encapsulation efficacy [47,48]. Solid particles such as silica particles have been proposed as alternative drug carriers that could potentially overcome such conventional problems. Silica particles with well-established synthesis methods are easily incorporated with other substances through surface modification, bioconjugation, and entrapment [49].

On the other hand, the use of biodegradable polymeric drug carriers, either synthetic or natural, shows great potential in controlled drug release by tuning their biodegradability. Of these, chitosan is the one of the most extensively studied - in particular, since it is composed of reactive hydroxyl and amine groups, it tends to adhere well to negatively charged surfaces, thus increasing cellular transport [50].

Because it is an antihypertensive drug, the systemic exposure of hydralazine will provoke problems related to blood pressure management in hypotensive trauma patients [51,52]. The use of our particulated system is capable of significantly reducing systemic exposure of drug since it is delivered to the intended site of action. In spite of its potent therapeutic effects reported here in vitro, our major challenge is to first, enhance the entrapment efficacy of hydralazine in the chitosan nanoparticle, and second, move the improved system to animal models of spinal cord and brain injury [12,19].

The first issue arises due to the existence of charge repulsion between cationic chitosan polymer (pKa = 6.5) and positively charged hydralazine (pKa = 7.1). In an attempt to enhance hydralazine encapsulation, two different kinds of polyanions were employed. Interestingly, the encapsulation of hydralazine was particularly dependent on the ionic interaction formed between chitosan and polyanions, where the amount of negatively charged groups of TPP and DS existed per mole was critical for hydralazine association. As a consequence, Chi-TPP formulation showed entrapment efficiency of 15.8% with slightly increased positively charged surface property, whereas Chi-DS exhibited an entrapment ratio of 23.5% with negative surface, characteristics as shown in (Figure 3c). In both cases, after entrapment of hydralazine, the nanoparticle formula showed some increase in diameter and zeta potential value, indicating the presence of drug through inter and intramolecular cross-linking. Both particles, Chi-TPP and Chi-DS, displayed similar trends in release behavior, where an initial burst release could likely be a result of the desorption of hydralazine (which is mostly attached on the surface of particles) and later progressive degradation of chitosan. Such a reduced release could be attributed to an increased viscosity resulting from the concentration ratio of chitosan and the polyanion, where a dense layer of chitosan might cause a greater density of crosslinking and consequently interfere with a swelling effect.

In summary, moving forward with this line of investigation requires exploring the stability of drug-loaded chitosan nanoparticles on the basis of pH or chemical crosslinking for a variety of compounds of interest to us. Various molecular weight chitosan samples or water-soluble chitosan molecules will be employed in this ongoing research. The second challenge requires a diversion towards toxicology studies to decipher any potential side effects or unforeseen problems associated with the chitosan particles or their cargo of hydralazine. Subsequently, we have much experience in the development of behavioral, physiological, and anatomical models of SCI and TBI, as well as access to veterinary clinical models of neurotrauma to move these notions forward.

## Materials and methods

### Preparation of chitosan nanoparticles: Chi-DS and Chi-TPP

Chitosan with 85% deacetylation degree and of medium weight (Chi, M.W. 200,000 Da), dextran sulfate (DS, M.W. 9,000 ~ 20,000 Da), and sodium tripolyphosphate (TPP, M.W. 367.8 Da) were purchased from Fluka/Sigma-Aldrich. Two kinds of chitosan particles were synthesized: Chi-DS and Chi-TPP. Briefly, Chi-DS was prepared by complexation of Chi and DS as described previously, where chitosan was dissolved at 0.10% (w/v) with a 1% aqueous acetic acid solution while DS was prepared in deionized water at the concentration of 0.5 mg/ml [26,28]. Equivalent volumes of chitosan and the DS solution were mixed by magnetic stirring at room temperature. Once the nanoparticle suspension started to form, the mixture was stirred for another 20 min. The formation of Chi-TPP nanoparticles was initiated by ionic gelation mechanism based on the interaction of cations and anions [27,33]. Chi-TPP nanoparticles were formed spontaneously when equal volume of Chi (1.75 mg/ml) and TPP (2 mg/ml) solution were prepared and stirred at room temperature. Hydralazine-loaded chitosan nanoparticles were then immediately prepared by incorporating equivalent volume of a Chi acidic solution (1.75 mg/ml) and an aqueous TPP solution (2 mg/ml) or aqueous DS solution (0.5 mg/ml) containing hydralazing (1 mg/ml) while stirring with a magnetic bar.

### Characterization of chitosan nanoparticles

Particle size and zeta potential measurements were carried out with a zeta-potential/particle size analyzer (Zetasizer). To begin, samples were diluted in deionized water and measured in an automatic mode. All measurements were performed in three ~ five repetitions. The morphology of chitosan nanoparticles was observed by transmission electron microscopy (JEOL 2000FX).

### Encapsulation and release of hydralazine from particles

The amount of hydralazine encapsulated in the chitosan nanoparticles was measured by UV spectrometry following centrifugation of the samples at 15000 rpm for 30 min. The difference between the total amount of hydralazine used for the formation of chitosan nanoparticle loaded with hydralazine and untrapped hydralazine in the supernatant solution was calculated to assess the efficiency of encapsulation.

To observe the release behavior of hydralazine from chitosan nanoparticles, a modified Krebs' solution (pH 7.2) that contained 124 mM NaCl, 2 mM KCl, 1.2 mM KH_2_PO_4_, 1.3 mM MgSO_4_, 2 mM CaCl_2_, 26 mM NaHCO_3 _was used. The release of hydralazine suspended in this Krebs' solution was observed as a function of the concentration of incorporated hydralazine. The released hydralazine was extracted at a different time-interval and centrifuged to permit measurement by UV spectroscopy. The concentration was then calculated by linear equation to determine the hydralazine release curve.

### Cell cultures

PC 12 cells with a density of 1 × 10^6 ^cells/mL were grown in Dulbecco's modified eagle's medium (DMEM; Invitrogen) supplemented with 12.5% horse serum, 2.5% fetal bovine serum, 50 U/ml penicillin, and 5 mg/ml streptomycin - at an incubator setting of 5% CO_2 _and 37°C. After trypsinization and centrifugation, cell pellets were resuspended in Hank's balanced salt solution (HBSS) for the exposure to acrolein and subsequent treatment with chitosan nanoparticles. Acrolein (100 μM) was prepared fresh daily in PBS solution. The Chi-TPP/Hy or Chi-DS/Hy suspensions were applied at a concentration of 20 μl/ml in medium with a delay of 15 min after the application of acrolein.

### Determining the integrity of cell membranes: The lactate dehydrogenase (LDH) exclusion assay

The reduction of nicotinamide adenine dinucleotide (NAD) by the presence of LDH results in the formation of a tetrazolium dye. The interpolation of the production of the dye can permit determination of the amount of LDH by standard spectrophotometric techniques. We use the LDH assay (Sigma) to assess cell membrane integrity after exposure of cells to the toxin acrolein. The amount of release of LDH from the cytoplasmic compartment to the supernatantis indicative of membrane permeability. Ordinarily, there would be little to no loss of LDH from intact cells. However in this assay, the necessary handling of cells results in some "background" loss of LDH which must be taken into account relative to the damage produced by acrolein-mediated attack on membranes. The background absorbance measured at 660 nm was subtracted from the reading at 492 nm. Cells were grown in 12-well plates at a density of 1 × 10^6 ^cells/well in HBSS were used for LDH release into the medium and determination of total LDH, respectively.

### MTT assay for cellular viability

Upon exposure to acrolein, the 3-(4,5-dimethylthiazol-2-yl)-2,5-diphenyltetrazolium bromide tetrazolium (MTT, Sigma) assay was used to determine the overall viability of the sample cells as it is an indicator of oxidative metabolism. The assay assesses the activity of a mitochondrial dehydrogenase enzyme which is detectable only from viable cells by the color change of tetrazolium rings from pale yellow MTT to dark blue formazan crystals. Quantification of these shifts was evaluated by spectrophotometric measurement. PC 12 cells were seeded in 12-well plates at 1 × 10^6 ^cells/well in HBSS. MTT reconstituted in PBS was added to each well. After incubation, formazan crystals were pelleted by centrifugation, and dissolved in a MTT solubilization solution. The absorbance was read at 550 nm minus the background at 660 nm.

### Cell mortality using the Live - Dead cell assay

To assess the impact of acrolein exposure on living cells, the determination of the number of live and dead cells in the total cell population was performed by the application of a two-color fluorescence assay (Biovision Inc.). This assay can selectively stain the live and dead cells using calcein AM (Cal AM) and ethidium homodimer (EthD-1). Cal AM typically crosses the cell membrane into living cells and consequently the cleaved calcein fluorophore is released which possess a strong green fluorescence with an emission wavelength of ~525 nm and an excitation wavelength of ~485 nm. In contrast, EthD-1 can only diffuse into dead or dying cells and produces a red fluorescence at ~625 nm when excited at ~525 nm. The test PC 12 cells were cultured in 12-well plate at 1 × 10^6 ^cells/well in HBSS, and subsequently incubated in Cal AM and EthD-1 for 5 minute. Using fluorescent microscopy, cell viability was calculated and expressed as a percentage.

### Measurement of reactive oxygen species (ROS) and lipid peroxidation (LPO)

The production of reactive oxygen species accelerates as a result of cell membrane damage which in turn drives LPO and toxic aldehyde production. Therefore a comparison of the levels of ROS and LPO following acrolein-exposure and post-treatment with chitosan nanoparticles provides clues for examining the state of cell deterioration in response to oxidative stress. The cultured cells were immersed in 1 mL of PBS with hydroethidium (HE, Invitrogen) at a final concentration of 1 μM for 10 min in the dark. Concentrations of ROS weredetermined by the chemi-luminescence assay based on the conversion of hydroethidine (HE) to ethidium (E^+^) in the presence of intracellular superoxide anion (O_2_^-^). Subsequently, samples were analyzed with epifluorescence on an Olympus BX61 microscope using a standard rhodamine cube (545/590 nm excitation/emission respectively), and quantified using Image J software (NIH) to measure the amount of ethidium bromide uptake. LPO was measured using a lipid hydroperoxide assay (Cayman Chem. Co.). Once cells were exposed to acrolein, the level of hydroperoxides was directly measured utilizing their redox reactions with ferrous ions. Subsequently, the absorbance of each sample was read at 500 nm using a spectrophotometer (SLT spectra plate reader). Lipid peroxidation was calculated and expressed as a percentage of control values.

### Statistical analysis

One-way ANOVA was used to determine the statistical significance between control and experimental groups using InStat software. Results are expressed as mean ± SD. P ≤ 0.05 was considered statistically significant.

## Competing interests

The authors declare they have no competing interests. There is no conflict of interest of any sort in the reporting of these data relative to any author.

## Authors' contributions

YC drafted the manuscript and performed the experiments. RS was helpful in the conduct of the experiments. RBB is the Principle Investigator and Director of the CPR and is responsible for all elements of the research. All authors read and approved final manuscript.
